# Effect of atorvastatin on glycaemia progression in patients with diabetes: an analysis from the Collaborative Atorvastatin in Diabetes Trial (CARDS)

**DOI:** 10.1007/s00125-015-3802-6

**Published:** 2015-11-17

**Authors:** Shona J. Livingstone, Helen C. Looker, Tahira Akbar, D. John Betteridge, Paul N. Durrington, Graham A. Hitman, H. Andrew W. Neil, John H. Fuller, Helen M. Colhoun

**Affiliations:** Diabetes Epidemiology Unit, Division of Population Health Sciences, University of Dundee, The Mackenzie Building, Kirsty Semple Way, Dundee, DD2 4BF Scotland UK; Department of Endocrinology and Diabetes, University College Hospital, London, UK; Cardiovascular Research Group, School of Biosciences, University of Manchester, Manchester, UK; Blizard Institute, Barts and the London School of Medicine, Queen Mary University of London, London, UK; Oxford Centre for Diabetes, Endocrinology and Metabolism, University of Oxford, Oxford, UK; Department of Epidemiology and Public Health, University College London, London, UK

**Keywords:** Atorvastatin, Cardiovascular disease, Glycaemia, HbA_1c_

## Abstract

**Aims/hypothesis:**

In an individual-level analysis we examined the effect of atorvastatin on glycaemia progression in type 2 diabetes and whether glycaemia effects reduce the prevention of cardiovascular disease (CVD) with atorvastatin.

**Methods:**

The study population comprised 2,739 people taking part in the Collaborative Atorvastatin Diabetes Study (CARDS) who were randomised to receive atorvastatin 10 mg or placebo and who had post-randomisation HbA_1c_ data. This secondary analysis used Cox regression to estimate the effect of atorvastatin on glycaemia progression, defined as an increase in HbA_1c_ of ≥0.5% (5.5 mmol/mol) or intensification of diabetes therapy. Mixed models were used to estimate the effect of atorvastatin on HbA_1c_ as a continuous endpoint.

**Results:**

Glycaemia progression occurred in 73.6% of participants allocated placebo and 78.1% of those allocated atorvastatin (HR 1.18 [95% CI 1.08, 1.29], *p* < 0.001) by the end of follow-up. The HR was 1.22 (95% CI 1.19, 1.35) in men and 1.11 (95% CI 0.95, 1.29) in women (*p* = 0.098 for the sex interaction). A similar effect was seen in on-treatment analyses: HR 1.20 (95% CI 1.07, 1.35), *p* = 0.001. The net mean treatment effect on HbA_1c_ was 0.14% (95% CI 0.08, 0.21) (1.5 mmol/mol). The effect did not increase through time. Diabetes treatment intensification alone did not differ with statin allocation. Neither baseline nor 1-year-attained HbA_1c_ predicted subsequent CVD, and the atorvastatin effect on CVD did not vary by HbA_1c_ change (interaction *p* value 0.229).

**Conclusions/interpretation:**

The effect of atorvastatin 10 mg on glycaemia progression among those with diabetes is statistically significant but very small, is not significantly different between sexes, does not increase with duration of statin and does not have an impact on the magnitude of CVD risk reduction with atorvastatin.

**Electronic supplementary material:**

The online version of this article (doi:10.1007/s00125-015-3802-6) contains peer-reviewed but unedited supplementary material, which is available to authorised users.

## Introduction

Although there is unequivocal evidence for the efficacy of statins in the prevention of cardiovascular disease (CVD), there continues to be intense debate about the adverse effects of statin therapy. There are concerns both about over-prescribing and conversely that patients warranting treatment are being deterred from treatment by inaccurate reports on adverse events [1, 2]. One potential adverse effect is an increased risk of diabetes. This increased risk was reported in Justification for the Use of Statins in Primary Prevention: an Intervention Trial Evaluating Rosuvastatin (JUPITER), a trial of rosuvastatin in people with elevated C-reactive protein, in which 3% of statin-treated and 2.4% of placebo-arm participants developed diabetes [3]. Subsequent meta-analyses of statin trials reported increased risks [4, 5] and observational studies also reported increased risks [6]. In 2012 the US Food and Drug Administration required that the drug safety label was amended to indicate an increased risk [7]. However, the aforementioned meta-analyses were not able to directly quantify the effect of statins on plasma glucose or on HbA_1c_ as these variables were not regularly measured in the trials examined. A recent study from Finland reported an even higher 46% increase in diabetes incidence associated with statins [8] accompanied by both loss of insulin sensitivity and secretion. However this observational study could be subject to substantial confounding by indication.

These data have also raised concerns about potential adverse effects on diabetes in those who already have diabetes at the start of statin therapy. Recently, a combined analysis of published treatment-arm summary data on HbA_1c_ from statin trials concluded that there was a small average effect on HbA_1c_ among those with diabetes [9]. However, this meta-analysis relied simply on published treatment-arm mean values at arbitrary points of follow-up and, as acknowledged by the authors, could not consider changes in diabetes drug use during follow-up. Thus, the estimates it used could be inaccurate and subject to competing risks bias. Furthermore, important questions remain about whether effects increase through duration of exposure. It is also unclear whether the reported effects on glycaemia reflect large effects occurring rarely in some susceptible subgroup of patients or small effects occurring commonly. Little is known about how any glycaemia effect relates to the degree of LDL-cholesterol (LDL-c) lowering or to other drug effects including liver function test changes. To address these questions individual-level data rather than meta-analyses are required.

Here we used individual-level data from a large primary prevention trial of atorvastatin 10 mg in people with existing diabetes at entry to directly test the effect of this statin on glycaemia progression as indexed by intensification of diabetes drug treatment or increasing HbA_1c_. We used a competing risks method that takes account of reduced CVD rates in those on statin therapy. We examined whether effects increased with duration of statin use and whether effects differed by sex. To explain possible mechanisms, we tested whether any effects were likely mediated by increased BMI and whether they related to the degree of LDL-c lowering or changes in liver function tests on treatment with statin. Finally we tested whether any adverse effect on glycaemia had an impact on the CVD risk reduction achieved with atorvastatin. Our analysis allows greater insight into the likely causality and mechanisms underlying any association and provides a practical insight allowing people with diabetes to weigh the benefits and risks of statin therapy given their condition.

## Methods

### Study population

The Collaborative Atorvastatin Diabetes Study (CARDS; ClinicalTrial.gov registration no. NCT00327418) has been described in detail previously [10]. In brief, CARDS was a multicentre, randomised, placebo-controlled trial in 2,838 patients with type 2 diabetes aged 40–75 years carried out in 132 centres in the UK and Ireland. Patients were randomised to receive either placebo (*n* = 1,410) or atorvastatin 10 mg (*n* = 1,428) daily. Study entrants had no documented previous history of CVD, an LDL-c concentration of 4.14 mmol/l or lower, a fasting triacylglycerol level of 6.78 mmol/l or less and an HbA_1c_ < 12% (<108 mmol/mol). The primary endpoint was time to first occurrence of acute coronary heart disease events, coronary revascularisation or stroke. The study was carried out in accordance with the Declaration of Helsinki and the guidelines on Good Clinical Practice, with each centre obtaining Local Research Ethics Committee approval following approval from the Multi-centre Research Ethics Committee. All patients gave fully informed written consent. Type 2 diabetes was defined using the 1985 WHO criteria [11]. The analysis population for this study consisted of 2,739 patients with at least one post-randomisation HbA_1c_ reading. This is a secondary analysis but before conducting this analysis all outcome measures and the analytical approach were pre-specified.

### Outcome measurement

We pre-specified a composite endpoint for glycaemia progression defined as an absolute increase in HbA_1c_ of at least 0.5% (5.5 mmol/mol) and/or an increase in the intensity of anti-glycaemic drug therapy. Intensity was defined by an ordinal variable set: 0 for neither oral drugs nor insulin; 1 for only one oral drug; 2 for two oral drugs; 3 for three or more oral drugs and 4 for insulin (regardless of the number of oral drugs). Non-insulin anti-glycaemic drugs observed in this study consisted mainly of metformin and sulfonylureas; the rare use of α-glucosidase inhibitors and the negligible use of thiazolidinediones and aldose reductase inhibitors was consistent with the era in which the study was conducted. The secondary pre-specified analysis was of HbA_1c_ change examined as a continuous variable.

HbA_1c_ was recorded at randomisation and then annually. We measured HbA_1c_ in whole blood containing fluoride oxalate using a Biorad Diamat high-pressure liquid chromatography analyser (Biorad, Hercules, CA, USA), with standards and controls supplied by the manufacturer. The upper limit of normal for the laboratory was 6.5% (48 mmol/mol). Aspartate aminotransferase (AST) or alanine aminotransferase (ALT) were measured using a Hitachi 747 autoanalyser, (Hitachi, Tokyo, Japan), with standards and controls recommended by the manufacturer. Serum cholesterol and triacylglycerol concentrations were measured by an automated enzymatic method. In samples not requiring ultracentrifugation (i.e. serum triacylglycerol ≤ 4 mmol/l) heparin-manganese precipitation of apo-B-containing lipoproteins was carried out on whole serum and HDL-cholesterol remaining in the supernatant fraction was measured by an enzymatic method. The LDL-c was then calculated using the Friedewald formula. BMI (kg/m^2^) was measured at baseline and at every visit thereafter. Waist–hip ratio was only captured at baseline. Concurrent medications were recorded at every visit.

### Statistical methods

Analyses were by intention-to-treat unless stated otherwise. All available data from randomisation were used, right censoring at the earliest of death, close-out date or last follow-up for cardiovascular morbidity. When we examined the effect of atorvastatin on glycaemic control the data were further right censored at the earlier of the last available HbA_1c_ measurement or loss to follow-up for concomitant medication. On-treatment, sensitivity analyses were further right censored after the date of first withdrawal from the study drug.

Cox regression models were fitted to the time to the binary outcome of glycaemia progression, stratified by major CVD status and adjusted for baseline age and diabetes duration with further adjustment for baseline lipids. Models were either adjusted for, or stratified by, sex. Effects on glycaemia progression were also analysed considering major CVD or death as competing risks using the method of Fine and Gray [12]. To test whether any effects of glycaemia progression were more pronounced with certain characteristics we tested for an interaction between the treatment effect on glycaemia progression and the characteristic concerned. To establish whether any treatment-associated progression was directly related to lipid lowering or possibly to other adverse effects, within-person changes in LDL-c and in AST, ALT, their ratio and serum creatinine kinase from randomisation to 6 months post-randomisation were calculated. The atorvastatin patients were dichotomised according to whether or not they showed a change over 6 months within the top tertile of change in LDL-c, AST, ALT as appropriate; each atorvastatin group was compared with all placebo patients in terms of subsequent glycaemia progression.

Maximum likelihood estimation was used to fit (repeated measures) linear mixed models to longitudinal data on HbA_1c_ and BMI using an unstructured covariance structure and nesting observations within patients and patients within trial sites. All models were adjusted by baseline age and diabetes duration and by linear and quadratic terms for time since randomisation. Models were fitted separately by sex, and for HbA_1c_ a model was also fitted to data on both sexes combined adjusting for sex. Models of HbA_1c_ change were further adjusted by baseline values of other baseline characteristics, and time-updated variables for BMI, anti-glycaemic drug intensity and having had a major CVD event. The effects of study drug on HbA_1c_ and BMI at 1 year post-randomisation were also assessed by simple ANCOVA models since at this time point there was less potential (compared with the full follow-up period) for interference by other factors such as changing diabetes drug therapy.

To understand the impact of these HbA_1c_ changes on CVD risk we first examined whether baseline or time-updated HbA_1c_ predicted CVD events Cox regression models. We then used tests for interaction to examine the effect of atorvastatin on CVD in patients with an above-median vs below-median change in HbA_1c_ at year 1. In these models, the entry time was the end of year 1 and those with a first CVD event in that first year were excluded.

All analyses were conducted using Stata/MP 11.1, StataCorp, College Station, TX, USA. Conventional *p* value thresholds of 0.05 were used for declaring statistical significance.

## Results

Of 2,838 randomised patients, 2,739 (96.5%) had at least one follow-up HbA_1c_ reading. The baseline characteristics of the analysis population were very similar by treatment arm, as shown in electronic supplementary material (ESM) Table 1. The mean baseline HbA_1c_ was slightly higher in the atorvastatin arm, by 0.05% (0.6 mmol/mol). The mean follow-up for HbA_1c_ change in the placebo group and atorvastatin group was 3.2 and 3.3 years, respectively; 95.3% of the participants had at least two readings and 76.0% and 47.4% had at least three or four follow-up readings, respectively. After censoring for loss to follow-up for concurrent medication or cardiovascular morbidity, the analysis population was reduced to 2,721 patients.

### Time-to-event analyses of glycaemia progression

By end of follow-up, glycaemia progression occurred in 996 of 1353 placebo patients (73.6%) and 1,069 of 1368 of atorvastatin patients (78.1%) (see Table 1 for sex-specific data). Adjusting for sex, baseline age, diabetes duration and HbA_1c_, and stratifying by major CVD status, the HR for the treatment effect was 1.18 (95% CI 1.08, 1.29) *p* < 0.001 in both sexes combined. Glycaemia progression occurred in 78.7% of men allocated atorvastatin and 73.3% allocated placebo (HR 1.22 [95% CI 1.10, 1.35], *p* < 0.001). Progression occurred in 77.0% and 74.4% of women allocated atorvastatin or placebo, respectively (HR 1.10 [95% CI 0.95, 1.29], *p* = 0.197; *p* = 0.289 for the diabetes by sex interaction).

**Table 1:** Cumulative incidence from baseline of increase in HbA_1c_ of ≥ 0.5% (5.5 mmol/mol) or intensification of anti-glycaemic drug therapy, or both, by treatment group

Time from baseline	Absolute increase in HbA_1c_ of ≥0.5%		Intensification of anti-glycaemic drug therapy		Composite of either endpoint	
	Placebo	Atorvastatin	Placebo	Atorvastatin	Placebo	Atorvastatin
Men						
1 year	38.0 (1.6), 873	47.7 (1.7), 887	0.8 (0.3), 873	1.6 (0.4), 887	38.8 (1.7), 873	48.9 (1.7), 887
2 years	55.7 (1.7), 844	61.0 (1.7), 854	7.1 (0.9), 844	6.6 (0.8), 854	60.1 (1.7), 844	64.4 (1.6), 854
3 years	65.7 (1.8), 665	70.2 (1.8), 681	14.3 (1.3), 665	14.4 (1.3), 681	69.5 (1.8), 665	74.9 (1.7), 681
4 years	69.8 (2.3), 404	76.0 (2.1), 417	31.4 (2.2), 404	33.9 (2.2), 417	78.7 (2.0), 404	84.2 (1.8), 417
Last follow-up	61.9 (1.6), 916	68.3 (1.5), 929	34.2 (1.5), 916	34.5 (1.5), 929	73.3 (1.5), 916	78.7 (1.3), 929
Women						
1 year	43.6 (2.5), 408	45.3 (2.4), 417	0.5 (0.3), 408	2.2 (0.7), 417	43.9 (2.5), 408	46.5 (2.4), 417
2 years	58.5 (2.5), 402	61.1 (2.4), 404	4.7 (1.1), 402	6.2 (1.2), 404	60.0 (2.4), 402	63.4 (2.4), 404
3 years	68.0 (2.7), 300	70.8 (2.6), 318	14.0 (2.0), 300	14.5 (2.0), 318	72.7 (2.0), 300	75.5 (2.4), 318
4 years	73.7 (3.2), 186	77.1 (2.9), 205	26.3 (3.2), 186	32.2 (3.3), 205	80.1 (2.9), 186	86.3 (2.4), 205
Last follow-up	66.4 (2.3), 437	67.0 (2.2), 439	28.8 (2.2), 437	37.1 (2.3), 439	74.4 (2.1), 437	77.0 (2.0), 439

Data are shown as cumulative incidence, % (SE), denominator

Data are based on 2,721 patients with 9,146 HbA_1c_ observations after right censoring for loss to follow-up for medications and cardiovascular morbidity

These findings were robust to further adjustment by the baseline lipid levels and intensity of diabetes therapy at baseline. In the competing risk analysis the sub-hazard of glycaemia progression was similar to the main model at HR 1.21 (95% CI 1.11, 1.32), *p* < 0.001. In a sensitivity analysis redefining progression as intensification of diabetes therapy or at least two consecutive HbA_1c_ readings at least 0.5% (5.5 mmol/mol) greater than baseline, a similar HR was observed (HR 1.18 [95% CI 1.07, 1.30], *p* = 0.001).

Overall glycaemia progression during follow-up was positively associated with younger age and lower baseline HbA_1c_, lower HDL-c concentration (*p* = 0.003) and higher triacylglycerol level (*p* = 0.002) but was not associated with LDL-c concentration (*p* = 0.720) or diabetes duration. However, there was no evidence that these or any other characteristics, including metabolic syndrome (*p* = 0.243) as previously defined, heightened the effect of atorvastatin on progression [13]. We found no evidence that the effect on glycaemia progression varied by tertile of post-treatment change in LDL-c (likelihood ratio test *p* value = 0.164), creatinine kinase (*p* = 0.241) or AST (*p* = 0.209) but found evidence that it did vary by change in ALT. Those in the top tertile for change from baseline in ALT had an HR of 1.39 (95% CI 1.19, 1.62) for the atorvastatin effect on glycaemia progression compared with an HR of 1.14 (95% CI 1.00, 1.62) for glycaemia progression associated with atorvastatin for those in the bottom tertiles for ALT change (*p* = 0.023 for the difference in treatment effect). This interaction was also present using the ALT:AST ratio.

See Table 1 and ESM Table 2 for a summary and details, respectively, of change in intensity of diabetes therapy from baseline. Overall there was no significant difference between treatment arms in the percentage of patients who progressed by at least one category of drug intensity with an HR of 1.09 (95% CI 0.96, 1.24), *p* = 0.184. Thus, most of the observed effect on glycaemia progression was ascertained through an effect on HbA_1c_; subsequent analyses focused on that but also adjusted for time-dependent changes in diabetes drug intensity.

### Effects on HbA_1c_ as a continuous trait

HbA_1c_ increase by at least 0.5% (5.5 mmol/mol) from baseline by treatment arm is shown in Table 1, and Table 2 shows median baseline and last follow-up level of HbA_1c_ and within-person changes from baseline over time by treatment arm. Overall the majority of participants in both treatment arms experienced such a rise at some point during follow-up (Table 1). However these increases were not always sustained and there was considerable fluctuation. Thus, using all available follow-up readings for HbA_1c_ in the mixed model, the median within-person change was much lower than 0.5% (5.5 mmol/mol) and the observed treatment effect, as detailed in Table 3, was 0.14 (95% CI 0.08, 0.21)% (1.5 [95% CI 0.8, 2.3] mmol/mol). Further adjustment (Models 2 and 3 in Table 3) for baseline lipids blood pressure, waist–hip ratio, baseline and time-updated BMI, diabetes drug intensity and time-updated CVD did not alter these findings. As shown, the difference between treatment arms was apparent at the median within-person changes. Thus, the treatment effect was consistent with a subtle shift in the change across the distribution rather than a large treatment effect occurring in a small set of patients.

**Table 2:** Median HbA_1c_ levels at baseline and within-person change from baseline to end of follow-up by treatment arm

Variable	Men		Women		Men and women	
	Placebo (*n =* 924)	Atorvastatin (*n =* 937)	Placebo (*n =* 437)	Atorvastatin (*n =* 441)	Placebo (*n*=1,361)	Atorvastatin (*n =*1,378)
Baseline HbA_1c_, %	7.6 (6.8–8.7)	7.7 (6.8–8.7)	7.6 (6.9-8.8)	7.8 (6.9–8.9)	7.6 (6.8–8.7)	7.8 (6.8–8.7)
Baseline HbA_1c_, mmol/mol	59.6 (50.8–71.6)	60.7 (50.8–71.6)	59.6 (51.9-72.7)	61.7 (51.9–73.8)	59.6 (50.8–71.6)	61.7 (50.8–71.6)
Last follow-up HbA_1c_, %	7.8 (7.0–8.7)	7.9 (7.1–9.0)	7.8 (7.1-8.9)	8.0 (7.1–8.9)	7.8 (7.1–8.8)	7.9 (7.1–9.0)
Last follow-up HbA_1c_, mmol/mol	61.7 (53.0–71.6)	62.8 (54.1–74.9)	61.7 (54.1-73.8)	63.9 (54.1–73.8)	61.7 (54.1–72.7)	62.8 (54.1–74.9)
Unadjusted within-person change in HbA_1c_, %	0.2 (−0.5–0.9)	0.3 (−0.5–1.1)	0.3 (-0.6-1.1)	0.2 (−0.6–1.0)	0.2 (−0.5–1.0)	0.2 (−0.5–1.1)
Unadjusted within-person change in HbA_1c_, mmol/mol	2.2 (−5.5–9.8)	3.3 (−5.5–12.0)	3.3 (-6.6-12.0)	2.2 (−6.6–10.9)	2.2 (−5.5–10.9)	2.2 (−5.5–12.0)

Data are median (IQR), unless otherwise stated

**Table 3:** Mixed model estimate of the effect of atorvastatin on change in HbA_1c_ using all available follow-up HbA_1c_ measures

Model	Net treatment effect		
	Men (*n*=1,861)	Women (*n*=878)	Men and women (*n*=2,739)
Model 1^a^			
%	0.18 (0.11, 0.26)	0.06 (−0.06, 0.18)	0.14 (0.08, 0.21)
mmol/mol	2.0 (1.2, 2.9)	0.6 (−0.7, 1.9)	1.5 (0.8, 2.3
Model 2 ^b^			
%	0.18 (0.10, 0.26)	0.06 (−0.06, 0.18)	0.14 (0.07, 0.21)
mmol/mol	2.0 (1.1, 2.9)	0.7 (−0.6, 2.0)	1.5 (0.8, 2.3)
Model 3 ^c^			
%	0.18 (0.10, 0.26)	0.08 (−0.04, 0.19)	0.14 (0.08, 0.21)
mmol/mol	2.0 (1.1, 2.8)	0.8 (−0.5, 2.1)	1.5 (0.8, 2.3)

Data are shown as % (95% CI) or mmol/mol (95% CI)

In the mixed models observations were nested within patient and trial site.

^a^Model 1 includes fixed effects of baseline HbA_1c_, age, diabetes duration, sex (when analysing both sexes together) and treatment group, as well as linear and quadratic terms for study time as random effects nested within individuals

^b^Model 2: Model 1 + intensity of anti-glycaemic drug therapy, systolic and diastolic blood pressure, total cholesterol, HDL-c and triacylglycerols

^c^Model 3: Model 2 + baseline waist–hip ratio and BMI, time-updated BMI, anti-glycaemic drug intensity and history of major CVD

**Fig. 1:**
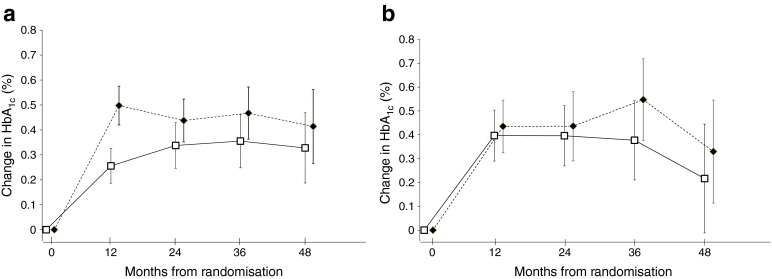
Mean within-person change in HbA_1c_ over time by treatment arm for men (**a**) and women (**b**): intention-to-treat analysis. Black diamonds, atorvastatin patients; white squares, placebo patients; capped lines, 95% CI. To convert values for changes in HbA_1c_ in % into mmol/mol, multiply by 10.929. Treatment effect in men: 0.18 (95% CI 0.11, 0.26). Treatment effect in women: 0.06 (95% CI −0.06, 0.08)

We also examined the net change at 1 year (during which time there was little change in intensity of drug treatment) and found similar effects. As shown in Fig. 1, the change in HbA_1c_ occurred mostly in the first year, with both placebo and atorvastatin groups showing increases and with no apparent worsening of the difference between the treatment arms through time.

### On-treatment sensitivity analyses

On-treatment analyses showed the same pattern as intent to treat. For glycaemia progression, the effect size was HR 1.20 (95% CI 1.07, 1.35), *p* = 0.001 (HR 1.25 [95% CI 1.09, 1.44], *p* = 0.002 in men and HR 1.13 [95% CI 0.92, 1.38], *p* = 0.240 in women; treatment–sex interaction *p* value = 0.332). For the effect on HbA_1c_ the effect size was HR 0.17 (95% CI 0.10, 0.25)% (HR 2.6 [95% CI 1.5, 3.6] mmol/mol), *p* < 0.001, being HR 0.23 (95% CI 0.14, 0.33)% (HR 1.9 [95% CI 1.1, 2.8] mmol/mol), *p* < 0.001, in men and HR 0.07 (−0.07,0.22)% (HR 0.8 [95% CI −0.8, 2.4] mmol/mol), *p* = 0.310, in women.

### Treatment effect on BMI

There was no evidence for an effect of atorvastatin on BMI (β coefficient 0.02 [95% CI −0.06, 0.10], *p* = 0.591). Including time-updated BMI as well as baseline BMI values in the analysis of treatment effect on HbA_1c_ did not explain any of the variation in HbA_1c_ change.

### Impact of HbA_1c_ change with treatment on the effect of atorvastatin on CVD

Baseline HbA_1c_ did not predict major CVD (HR per percentage unit adjusted for age and sex 1.08 [95% CI 0.98, 1.19], *p* = 0.110). Nor did HbA_1c_ attained at 1 year predict subsequent major CVD (HR 1.08 [95% CI 0.96, 1.21], *p* = 0.214). There was no evidence of an interaction between treatment and change in HbA_1c_ dichotomised below or above the overall median change. The HR for the effect of atorvastatin on CVD was 0.47 (95% CI 0.29, 0.77) in those with below-median change in HbA_1c_ at 1 year vs 0.63 (95% CI 0.41, 0.97) in those with above-median change (interaction *p* value = 0.229).

## Discussion

This analysis of statin effect on glycaemia within a diabetes population finds evidence for an effect of atorvastatin 10 mg daily on glycaemia progression. However the effect is modest, with a net increase in HbA_1c_ of 0.14% (1.5 mmol/mol) by the end of follow-up. As expected there was considerable variation between patients in the net within-person change in HbA_1c_ over time, with the effect of atorvastatin being very slight in relation to this variance in within-person change. This effect of atorvastatin on glycaemia was most apparent in the first year following initiation of therapy and, reassuringly, did not get worse through time. The effect seems to be subtle and to be a common effect rather than a large effect that occurs in a particular subgroup of patients. The only correlate of the effect was the rise in ALT during statin therapy. Importantly, we did not find any evidence that such effects on glycaemia have any material impact on the large preventive effect of atorvastatin on CVD in people with diabetes. Quantification of the glycaemic effect in diabetes is important for helping people already with diabetes, and their doctors, to arrive at an informed decision on risks and benefits of statin therapy. Of course, when making such decisions, other issues such as absolute risk of CVD, co-morbidities and other potential side effects need to be taken into consideration.

The effect on glycaemia we observed is consistent with the findings of previous meta-analyses showing increases in incident diabetes with statins [5]. With regard to effects on measures of glycaemia there are fewer robust analyses. Small short-term intervention studies reported increases in insulin and plasma glucose or glycated albumin in hyperlipidaemic patients without diabetes [14, 15]. More recently, Erquo et al [9] combined treatment-arm means from published trials of those with diabetes, including unadjusted group means at 4 year follow-up, which we included as a safety analysis in the main results paper from CARDS [10]. As acknowledged by Erquo et al, since it was not an individual-level analysis they could not assess whether effects were robust to alterations in diabetes drugs throughout the trials, nor could they assess whether or not effects increased through duration of exposure. Also, as they did not have any data on the distribution of the statin effect it remained unclear whether these average effects reflect subtle common effects or rarer extreme effects [9].

With regard to the mechanism of this effect, the most recent meta-analysis of the effect of statins on incident diabetes was accompanied by a Mendelian randomisation analysis showing that in large association studies participants with LDL-c-lowering single-nucleotide polymorphisms in the 3-hydroxy-methylglutaryl-CoA reductase gene (with an allele effect on LDL-c of 0.06 mmol/l on average) had slightly higher rates of type 2 diabetes (OR per allele 1.02 [95% CI 1.00, 1.05]). These data were interpreted to prove a direct relationship between LDL-c lowering with statins and increased diabetes risk [5]. In contrast we did not find any relationship with degree of LDL-c lowering in this study. Furthermore, in that analysis BMI at follow-up was increased with statin therapy in active vs placebo trials but, in contrast, we found no effect of atorvastatin on BMI despite having detailed data on this outcome. We did find some evidence that those with a greater increase in ALT following treatment were more susceptible. The METSIM (Metabolic Syndrome in Men) population-based study did report significant increases in 2 h glucose and glucose area under the receiver operating characteristic curve of an OGTT at follow-up for men without diabetes at baseline [8]; reduced insulin sensitivity and secretion, along with a 46% increased risk of incident diabetes adjusted for baseline were also found. However, these differences could reflect confounding by indication in this observational analysis where statin recipients clearly already differed substantially from non-recipients in characteristics at baseline, before the onset of diabetes [8].

We examined whether there are specific subgroups of people with diabetes who are most susceptible to the glycaemic effect of statins. However, we did not find any characteristics that delineated particular individuals as being susceptible to this effect. In contrast, in an analysis of atorvastatin trials in those without diabetes at initiation of therapy, those who developed new-onset type 2 diabetes were more likely to have hypertension at baseline, to be taking beta blockers and to have higher fasting glucose, BMI, white blood cell count, systolic and diastolic blood pressure, total cholesterol/ HDL-cholesterol ratio, triacylglycerol level, and lower HDL-cholesterol [16]. In the JUPITER trial, the increased risk of diabetes with statin therapy was only observed in those with at least one diabetes risk at entry [17]. Those with one or more major diabetes risk factors (*n* = 11,508) were more likely to be women. An observational analysis from the Women’s Health Initiative reported a 1.7-fold increase in incident diabetes in postmenopausal women after starting statins [6]. While adjustment for confounding did not reduce this risk, the potential for residual confounding to affect such observational studies remains. In contrast to these findings, we found no evidence that the effect was greater in women than in men. Sex was not associated with new-onset type 2 diabetes in an analysis of atorvastatin trials [16].

In this study we were able to examine whether the effect of atorvastatin on glycaemia has any substantial impact on its ability to prevent CVD and we conclude that it does not. The HRs are of the same order of magnitude whether glycaemia progressed or not. Our data on the relative importance is consistent with the conclusions reached by Ridker et al [17] in their analysis of JUPITER trial data on those without diabetes at initiation of therapy. They concluded that the cardiovascular and mortality benefits of statin therapy exceeded the diabetes hazard, including in participants at high risk of developing diabetes.

A useful aspect of our analysis that has remained unresolved in the meta-analyses so far is the role of competing risks. Previous meta-analyses were not able to assess the potential role of competing risk effects as individual-level data are needed for this. Here, such formal competing risk analyses showed that the glycaemia effect could not be attributed to this. Also the overall treatment effect is mainly driven by the change occurring in year 1. Thus, adjusting for competing risks of CVD or death has little effect on the findings.

The limitations of this study are that only atorvastatin rather than other statins and only one dose, 10 mg, was evaluated. Previous meta-analyses in individuals without diabetes have suggested that there is a dose–response effect [5, 18]. Nonetheless, a dose of 10 mg is very commonly used in diabetes; since we have shown that many patients will achieve substantial LDL-c lowering with this dose our data have wide relevance. Additionally, the sample size precludes precise quantification of effects within subgroups. Although in the analysis of effects on HbA_1c_ we did adjust for time-updated changes in intensity of diabetes medications, based on the number of drugs being used, we did not adjust for dose changes within a specific category of oral drugs. However, since the estimate of effect really did not change at all, even when adjusting for time-updated change in diabetes drug intensity, it seems unlikely that it would change in a major way with the more subtle adjustment for drug dose.

Another limitation is that we did not have retinal photograph data to enable us to quantify any effects on retinopathy, which is one of the diabetes-related complications most susceptible to glycaemia.

In conclusion, although the effect of atorvastatin 10 mg on HbA_1c_ and on glycaemia progression in patients with diabetes is statistically significant, it is very small and it does not increase with duration of statin use or have an impact on the substantial reduction in CVD risk with atorvastatin.

## Electronic supplementary material

ESM Table 1 (PDF 19 kb)

ESM Table 2 (PDF 28 kb)

## Abbreviations

AST: Aspartate aminotransferase
ALT: Alanine aminotransferase
CARDS: Collaborative Atorvastatin Diabetes Study
CVD: Cardiovascular disease
JUPITER: Justification for the Use of Statins in Primary Prevention: an Intervention Trial Evaluating Rosuvastatin
LDL-c: LDL-cholesterol

## References

[1] Armitage J, Baigent C, Collins R (2014). Misrepresentation of statin safety evidence. Lancet.

[2] Godlee F (2014). Statins and the BMJ. BMJ.

[3] Ridker PM, Danielson E, Fonseca FA (2008). Rosuvastatin to prevent vascular events in men and women with elevated C-reactive protein. N Engl J Med.

[4] Sattar N, Preiss D, Murray HM (2010). Statins and risk of incident diabetes: a collaborative meta-analysis of randomised statin trials. Lancet.

[5] Swerdlow DI, Preiss D, Kuchenbaecker KB (2014). HMG-coenzyme A reductase inhibition, type 2 diabetes, and bodyweight: evidence from genetic analysis and randomised trials. Lancet.

[6] Culver AL, Ockene IS, Balasubramanian R (2012). Statin use and risk of diabetes mellitus in postmenopausal women in the Women’s Health Initiative. Arch Intern Med.

[7] U.S. Food and Drug Administration (2012) FDA Drug safety communication: important safety label changes to cholesterol-lowering statin drugs. Available from http://www.fda.gov/Drugs/DrugSafety/ucm293101.htm, accessed 6 October 2015

[8] Cederberg H, Stancakova A, Yaluri N, Modi S, Kuusisto J, Laakso M (2015). Increased risk of diabetes with statin treatment is associated with impaired insulin sensitivity and insulin secretion: a 6 year follow-up study of the METSIM cohort. Diabetologia.

[9] Erqou S, Lee CC, Adler AI (2014). Statins and glycaemic control in individuals with diabetes: a systematic review and meta-analysis. Diabetologia.

[10] Colhoun HM, Betteridge DJ, Durrington PN (2004). Primary prevention of cardiovascular disease with atorvastatin in type 2 diabetes in the Collaborative Atorvastatin Diabetes Study (CARDS): multicentre randomised placebo-controlled trial. Lancet.

[11] World Health Organization (1985) Diabetes mellitus. Report of a WHO Study Group. (Technical Report Series 727). World Health Organization, Geneva 3934850

[12] Fine J, Gray R (1999). A proportional hazards model for the subdistribution of a competing risk. J Am Stat Assoc.

[13] Alberti KG, Zimmet P, Shaw J (2006). Metabolic syndrome--a new world-wide definition. A Consensus Statement from the International Diabetes Federation. Diabet Med.

[14] Koh KK, Quon MJ, Han SH, Lee Y, Kim SJ, Shin EK (2010). Atorvastatin causes insulin resistance and increases ambient glycemia in hypercholesterolemic patients. J Am Coll Cardiol.

[15] Thongtang N, Ai M, Otokozawa S (2011). Effects of maximal atorvastatin and rosuvastatin treatment on markers of glucose homeostasis and inflammation. Am J Cardiol.

[16] Waters DD, Ho JE, DeMicco DA (2011). Predictors of new-onset diabetes in patients treated with atorvastatin: results from 3 large randomized clinical trials. Am J Cardiol.

[17] Ridker PM, Pradhan A, MacFadyen JG, Libby P, Glynn RJ (2012). Cardiovascular benefits and diabetes risks of statin therapy in primary prevention: an analysis from the JUPITER trial. Lancet.

[18] Preiss D, Seshasai SR, Welsh P (2011). Risk of incident diabetes with intensive-dose compared with moderate-dose statin therapy: a meta-analysis. JAMA.

