# Supplementary material for: Effect of atorvastatin on glycaemia progression in patients with diabetes: an analysis from the Collaborative Atorvastatin in Diabetes Trial (CARDS)
Source: Diabetologia. 2015 Nov 17;59:299–306. doi: 10.1007/s00125-015-3802-6 (PMC4705133; doi:10.1007/s00125-015-3802-6)
Supplement: Supplementary file 1 — (PDF 19 kb) [file 125_2015_3802_MOESM1_ESM.pdf]

Electronic Supplementary Material (ESM)

ESM Table 1. Baseline characteristics by treatment group

Characteristic	Placebo[a] (*n* = 1361)	Atorvastatin[a] (*n* = 1378)	Age, sex adjusted P-value[b]
Female	32.1 (1.3)	32.0 (1.3)	0.942
Age, years	61.8 (8.0)	61.5 (8.2)	0.442
Diabetes duration, years	7.7 (6.3)	7.9 (6.4)	0.394
HDL-c, mmol/L	1.41 (0.34)	1.39 (0.32)	0.193
Triacyglycerol, mmol/L	1.94 (1.08)	1.95 (1.09)	0.846
LDL-c, mmol/L	3.02 (0.7)	3.04 (0.72)	0.381
HbA_{1c}, %	7.81 (1.39)	7.86 (1.41)	0.355
HbA_{1c}, mmol/mol	61.8 (15.2)	62.4 (15.4)	0.355
BMI, kg/m^2	28.9 (3.5)	28.7 (3.6)	0.129
Metabolic syndrome	54.6 (1.4)	54.6 (1.3)	0.914
Anti-glycaemic drug therapy			
None	16.1	15.0	-
Metformin	13.7	13.6	-
Sulphonylurea	22.3	21.5	-
Other monotherapy[c]	0.3	0.4	-
2 orals	26.3	27.1	-
3 orals	2.4	2.4	-
Insulin	18.9	20.2	0.956[d]

[a] Mean (standard deviation) and percent (standard error of percent) are shown for continuous and binary variables respectively. [b] P-value from logistic regression of treatment arm adjusted by baseline age and sex where relevant.
[c] Other monotherapy consists mainly of alpha glucosidase inhibitors (111 of 131) with only 12 and 8 patients reporting thiazolidinediones and aldose reductase inhibitors respectively. [d] P-value for 7 level categorical variable.
