# Supplementary material for: Effect of atorvastatin on glycaemia progression in patients with diabetes: an analysis from the Collaborative Atorvastatin in Diabetes Trial (CARDS)
Source: Diabetologia. 2015 Nov 17;59:299–306. doi: 10.1007/s00125-015-3802-6 (PMC4705133; doi:10.1007/s00125-015-3802-6)
Supplement: Supplementary file 2 — (PDF 28 kb) [file 125_2015_3802_MOESM2_ESM.pdf]

Electronic Supplementary Material

ESM Table 2. Anti-glycaemic drug use at last follow-up for HbA_{1c} compared to baseline drug treatment

	At last follow-up, number of patients (% of number in baseline category) [a]								
	None	Metformin	SU[b]	Other oral monotherapy	2 orals	≥3 orals	Insulin	Total	% of Grand Total
Placebo									
None	114 (54.0)	40 (19.0)	34 (16.1)	1 (0.5)	19 (9.0)	1 (0.5)	2 (1.0)	211 (100)	15.6
Metformin	21 (11.3)	87 (46.8)	8 (4.3)	3 (1.6)	61 (32.8)	1 (0.5)	5 (2.7)	186 (100)	13.8
SU	26 (8.6)	13 (4.3)	123 (40.5)	2 (0.7)	100 (32.9)	6 (2.0)	34 (11.2)	304 (100)	22.5
Other oral monotherapy	0 (0)	0 (0)	0 (0)	1 (25.0)	3 (75.0)	0 (0)	0 (0)	4 (100)	0.3
2 orals	15 (4.2)	33 (9.2)	32 (8.9)	4 (1.1)	166 (46.4)	15 (4.2)	93 (26.0)	358 (100)	26.5
≥3 orals	0 (0)	1 (3.0)	0 (0)	0 (0)	8 (24.2)	12 (36.4)	12 (36.4)	33 (100)	2.4
Insulin[c]	19 (7.4)	7 (2.7)	0 (0)	1 (0.4)	0 (0)	0 (0)	230 (89.5)	257 (100)	19.0
Total	195 (14.4)	181(13.4)	197 (14.6)	12 (0.9)	357 (26.4)	35 (2.6)	376 (27.8)	1353 (100)	100

	At last follow-up, number of patients (% of number in baseline category) [a]								
	None	Metformin	SU[b]	Other oral monotherapy	2 orals	≥3 orals	Insulin	Total	% of Grand Total
Atorvastatin									
None	94 (46.8)	47 (23.4)	33 (16.4)	1 (0.5)	18 (9.0)	1 (0.5)	7 (3.5)	201 (100)	14.7
Metformin	20 (10.8)	93 (50.0)	5 (2.7)	3 (1.6)	56 (30.1)	0 (0)	9 (4.8)	186 (100)	13.6
SU	34 (11.6)	13 (4.4)	110 (37.5)	4 (1.4)	96 (32.8)	9 (3.1)	27 (9.2)	293 (100)	21.4
Other oral monotherapy	0 (0)	1 (20.0)	0 (0)	2 (40.0)	0 (0)	0 (0)	2 (40.0)	5 (100)	0.4
2 Orals	13 (3.5)	35 (9.4)	30 (8.0)	5 (1.3)	154 (41.3)	17 (4.6)	119 (31.9)	373 (100)	27.3
≥3 Orals	1 (3.0)	0 (0)	0 (0)	0 (0)	5 (15.2)	8 (24.2)	19 (57.6)	33 (100)	2.4
Insulin[c]	24 (8.7)	15 (5.4)	0 (0)	1 (0.4)	0 (0)	1 (0.4)	236 (85.2)	277 (100)	20.3
Total	186 (13.6)	204 (14.9)	178 (13.0)	16 (1.2)	329 (24.1)	36 (2.6)	419 (30.6)	1368 (100)	100

[a] N=2721 patients evaluable for glycaemia progression and major cardiovascular event status. Other oral monotherapy consists mainly of alpha-glucosidase inhibitors and very few thiazolidinediones or aldose reductase inhibitors. [b] SU, sulphonylureas. [c] Insulin with or without metformin, SU or other oral anti glycaemic drugs.
